# Investigation of craquelure patterns in oil paintings using precise 3D morphological analysis for art authentication

**DOI:** 10.1371/journal.pone.0272078

**Published:** 2022-07-28

**Authors:** Soojung Kim, Sang Min Park, Seongjin Bak, Gyeong Hun Kim, Chang-Seok Kim, Joonja Jun, Chang Eun Kim, Kyujung Kim

**Affiliations:** 1 Department of Cogno-Mechatronics Engineering, Pusan National University, Busan, Republic of Korea; 2 Department of Fine Arts, Pusan National University, Busan, Republic of Korea; 3 Department of Optics and Mechatronics Engineering, Pusan National University, Busan, Republic of Korea; William & Mary, UNITED STATES

## Abstract

The development of scientific technology for art authentication has elicited multidimensional evidence to distinguish forgeries from original artwork. Here, we analyzed the three-dimensional morphology of cracks that contain information, such as the painting features of artworks, using optical coherence tomography. The forgeries were produced by an expert from original oil paintings with cracks that occur owing to paint drying, canvas aging, and physical damage. Parameters, such as shape, width, and depth, were compared based on the cross-sectional images of the original and fake cracks. The original cracks were rectangular and inverted, but the fake cracks were relatively simple inverted triangles. The original cracks were as deep as the thickness of the upper layer and mostly were “thin/deep” or “wide/shallow”. The fake cracks were observed to be “’thin/shallow” or “wide/deep”. This study aims to improve the understanding of crack characteristics and promote the development of techniques for determining art authenticity.

## 1. Introduction

The volume and value of transactions in the international art market have been increasing over the years [1–3]. General interest in artworks has progressively increased in recognition of their financial and cultural value [4, 5]. Simultaneously, art forgery scandals have occurred for monetary gain. Consequently, the reliability of the global art market has diminished, and economic turbulence may occur in global art sales [6]. Counterfeit technologies have grown rapidly, and there are cases in which authentication tests cannot be judged to be reliable, even when using various technologies. To sustain the artistic activities of artists and transactions in the art market, a specialized system needs to be established to distinguish between authentic and forged artworks. Two methods can be used to discriminate counterfeit works: a judging process with expert insight through observations with the naked eye [7] and scientific assessments using analytical techniques [8–10]. In the first method, the appearance of artworks is visually inspected for pigments, paint aging, canvas aging, and cracking generated on the surface of artworks. In the second method, scientific techniques, such as bright-field microscopy [11], scanning electron microscopy [12], transmission electron microscopy [13], infrared imaging [14, 15], and ultraviolet imaging [16], are used to examine the artworks.

Recent studies have shown that cracks on painted surfaces contain information on environmental factors, the proportions of pigments, humidity, and the unique characteristics of the artist’s painting technique, making it possible to judge the authenticity of artworks [17–20]. The physical [17, 21, 22] and visual [20, 23] characteristics of cracks on the painted surface have been investigated to analyze cracks. When studying the physical characteristics of cracks, some pigment layers of the artworks need to be extracted occasionally, and the internal and external structures of paintings cannot be examined physically without irrevocable damage to the original piece. However, important information can be obtained through visual analysis, such as the characteristics of the painting and the types of cracks [9, 21]. The overall shape of the crack in the top view can be detected with the naked eye and can be enlarged significantly using a microscope that provides high-magnification images. Natural cracks were successfully distinguished from forged cracks using an ordinary microscope [24, 25]. The general optical microscope can quickly analyze cracks distributed over the entire painted surface, but only the top surface of the cracks can be observed. However, cracks on painted surface are generated with three-dimensional geometrical characteristics by applying the various factors, such as materials, techniques, and the environmental variables [18, 20, 26]. Combining emerging vertical analysis with conventional horizontal analysis can improve the performance and accuracy of the artwork authentication process.

In this study, we focus on the cross-sectional analysis of cracks on a painted surface using an optical coherence tomography (OCT) system for an accurate estimation of the crack dimensions (width, depth, and morphology) [27–29]. OCT laser technology has been utilized to investigate the pigment layers of artworks without inflicting damage to them and has proven to be highly effective for artwork analysis [30–35]. We investigated differences between natural and artificial cracks forged by counterfeiters to provide a basis for authenticity through OCT analysis. The suggested OCT analysis method can be used as an indirect basis and cannot be an absolute answer for determining the authenticity of artworks because the shape of the crack may change owing to various reasons, such as paint type, characteristics, and surface treatment of the emulsified work. In conclusion, this study is expected to advance understanding characteristics of the crack surface and contribute significantly to the determination of art authenticity.

## 2. Materials and methods

### 2.1 Experimental process of OCT analysis

**Fig 1:**
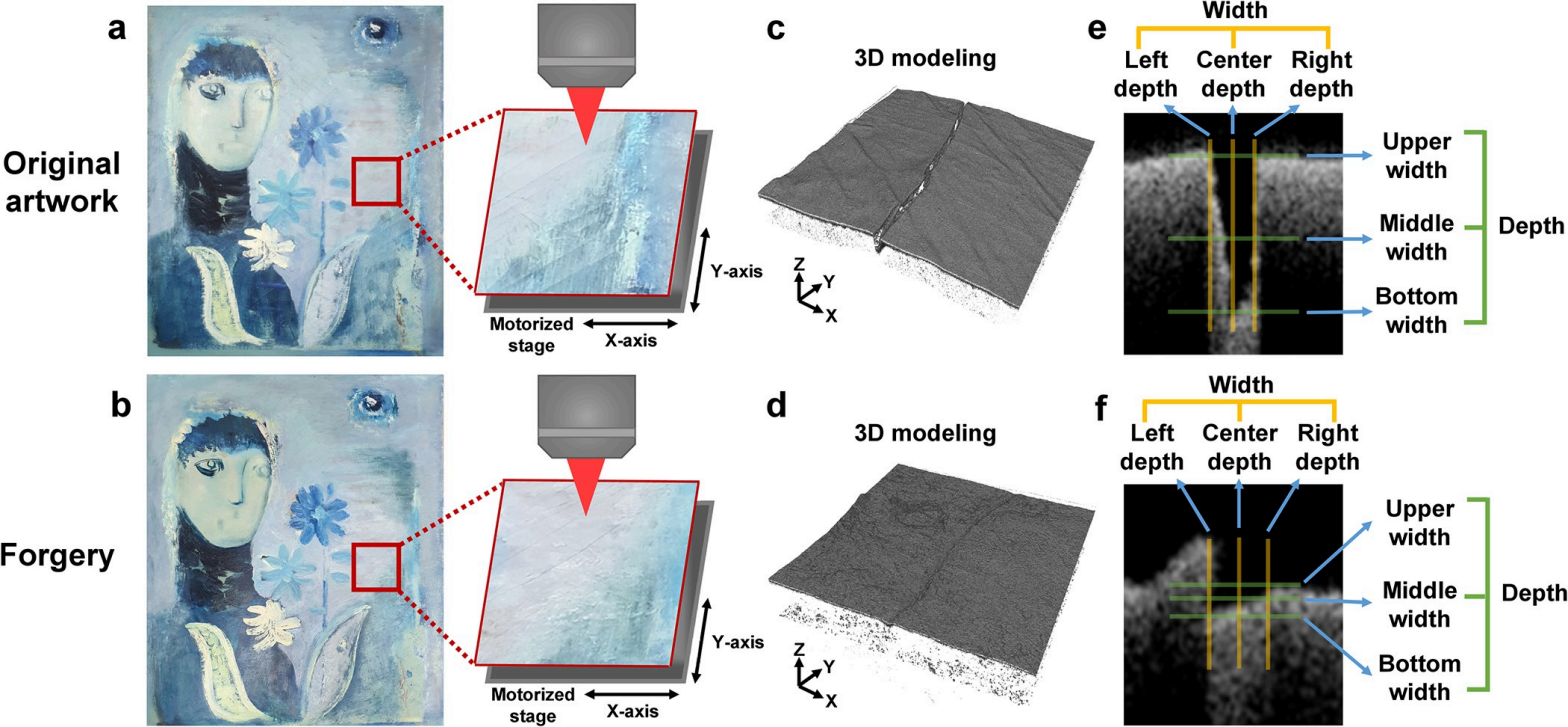
Measurement of the three-dimensional shape of cracks on the surface of artworks through OCT and analysis of the evidence that can be used for authenticity judgment. **(a, b)** OCT system set-up to measure the crack area of the artwork. This system can fix artworks on the stage to prevent unwanted movement while the artwork is being evaluated. The stage moves them in units of at least 10 μm along the XY-axis and the focused beam on the crack area to accurately measure the desired area. **(c, d)** Acquired 3D data of art objects and **(e, f)** cross-sectional images extracted to compare the width and depth parameters of the original and forged artworks.

A schematic depicting the authenticity assessment process for artworks based on OCT technology is shown in **Fig 1**. Three artworks were selected for the OCT analysis according to the time of production, the artist’s expertise, and the characteristics of the cracks. Natural cracks occur owing to various causes such as physical and chemical reactions at the painted surface for each work, and these cracks were analyzed using OCT technology **(Fig 1A)**. An art restoration expert, who created two of the artworks, produced forgery works for each of his two artworks and the one of other artworks with permission from the original artist **(Fig 1B)**.

Before analyzing the paintings using OCT technology, several cracks that represent the characteristics of the artworks were selected from the cracks in each original work. In the counterfeit works, artificial cracks made at locations similar to those in the original work were selected. OCT scanning was performed by selecting a measurement area of 2 mm × 2 mm × 1.7 mm at the crack position on the artwork surface **(Fig 1C and 1D)**. Subsequently, 3D modeling data containing information on the depth, width, cross section, and shape of the cracks were obtained, and a cross-sectional image of the middle plane of the cracks was extracted as representative for the quantitative analysis of crack morphologies. In the cross-sectional image, various parameters, such as width, depth, and shape, were measured according to the morphological diversity and complexity of the cracks. In particular, the width and depth were subdivided into three categories to analyze the crack characteristics in as much detail as possible **(Fig 1E and 1F)**. Based on the data obtained in this manner, the characteristics of the cracks were classified and analyzed according to the cause of the crack, and the foundation for the authenticity of the artwork was established. The width and depth of crack may vary depending on the cross-sectional image extracted from the three-dimensional image of crack Therefore, the quantified values presented in this study can be used as supplementary data to explain the characteristics of the cracks.

### 2.2 Experimental set-up of the OCT system

The OCT system was constructed using a Michelson interferometer with a beam splitter. In this setting, a spectral domain OCT system was selected for high-speed data acquisition **(Fig S1 in S1 File)**. The developed OCT system generates a circularly polarized laser operating at a wavelength of 930 nm. The penetration depth of the laser on the painted surface of the artworks could be reduced compared with the longer wavelength of the laser source; however, the OCT system using a laser with a short wavelength had a higher imaging performance with a better resolution for accurate crack detection. In this study, the experimental OCT system could produce an excellent longitudinal resolution of 8 μm and a lateral resolution of 7 μm in air and a measuring depth range of 1.7 mm from the surface. After laser emissions were transmitted through the beam splitter, they were reflected from the two scanning mirrors and injected onto the surface of the artwork with an objective lens. Interference signals were produced by combining the reflected optical signals from the artwork surface with the reference signals. 3D reconstructed images were generated with accurate dimensional and morphological characteristics of the surface cracks.

### 2.3 Preparation of original and forged artworks

**Fig 2:**
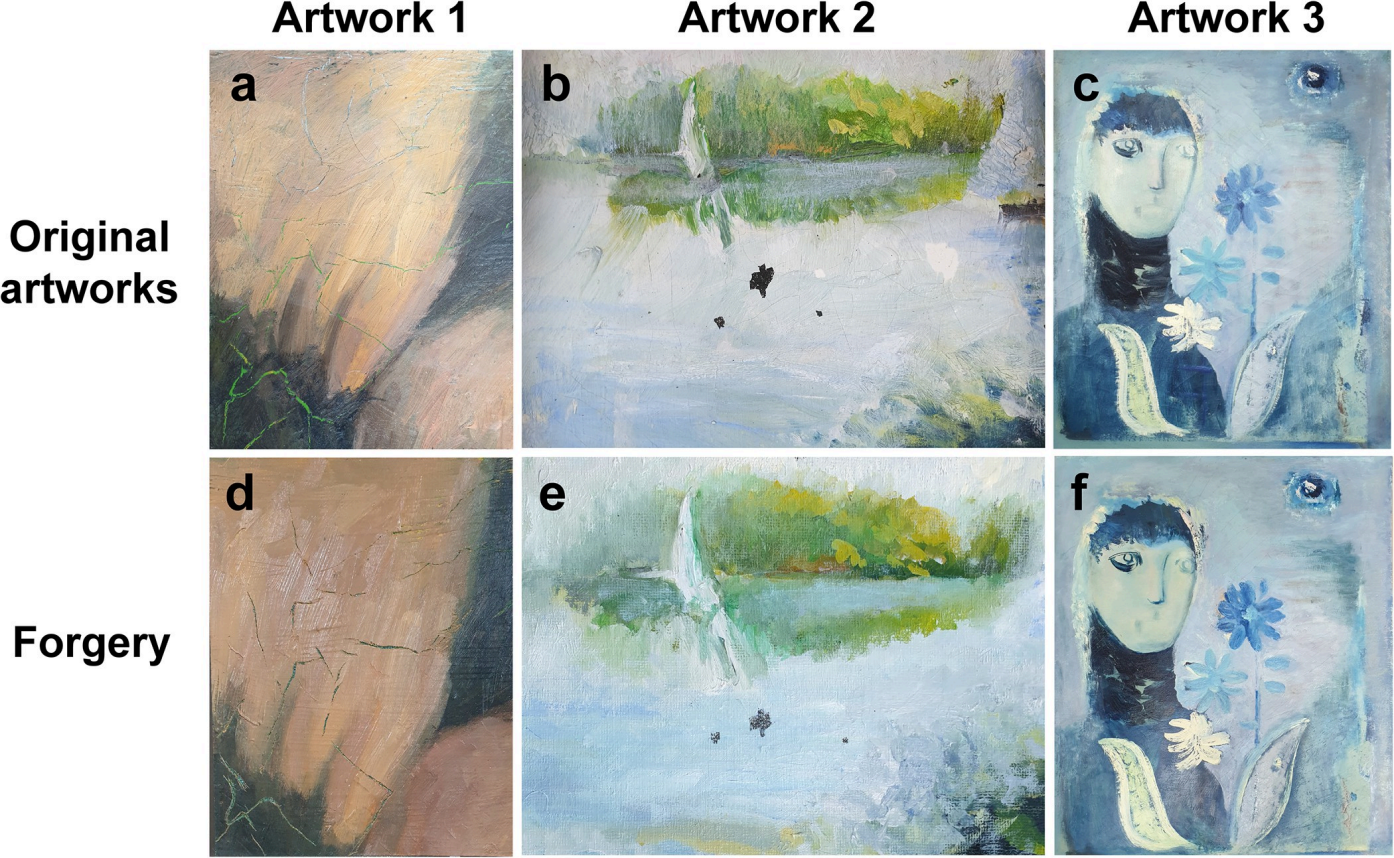
Original oil painting **(a, b, and c)** and counterfeit works **(d, e, and f).** They were prepared for an analysis of the three-dimensional forms of cracks used for authenticity identification of art works. The forgeries were completed by checking the color combination of the original work, the artist’s brushstroke style, and the thickness of the paint layer as much as possible.

The three artworks analyzed in this study were oil paintings by Korean artists who agreed to the contents of this study **(Fig 2)**. All the paintings used in this study were stored without any light or dust at a temperature of 25°C and a humidity of 40% to prevent the deterioration of their surfaces. In addition, these artworks were stored in a chemical-free space and were fixed to an easel to avoid contact with the floor and surrounding objects. As cracks are likely to occur in old works, art works that were at least 30 years old were selected. Two of these artworks were created by Eunyeong Ko, who specializes in oil paintings and art restoration **(Fig 2A and 2B)**. The third painting was “*figure*” (45 cm × 38 cm), oil painting created by Joonja Jun in 1964 **(Fig 2C)**. Joonja Jun is an acclaimed contemporary Korean artist who was selected as a recommended artist for the National Exhibition and won three special awards and 13 awards at the Korean National Art Exhibition held from 1949 to 1981. A major theme of her work is humans and festivals. By making full use of intense gesture drawing and action painting, the artist pursues a fundamental inquiry into painting.

With permission from the original artist, a restoration expert produced counterfeit works of the original works using the appropriate oil paint and medium after observing the style, color, and cracks of the original work through the naked eye and a magnifying lens **(Fig 2D–2F)**. The art restoration expert could accurately understand the color combination of the original work, the thickness of the paint layer, the texture of the surface, and the painting style because these features are essential for restoring damaged areas in artworks. The art restoration specialist used appropriate oil paints and media to replicate the colors and cracks as accurately as possible.

The methods for the creation of artificial cracks on the surface of artworks can be broadly classified into two types: ⅰ) physical processes such as cutting with knives and the bending deformation of painted layers, and ⅱ) chemical processes using a crackling medium and crackle paste. As dry cracks tend to be wider than aging cracks [17], cracks were created using sharp knife tools with the advantage of adjusting the location, network pattern, width, and length of cracks as much as possible to resemble the original cracks. The cracks were artificially created by drawing them on the surface of the artwork with physical force using various sharp knives, and the thickness of the cracks was adjusted by adjusting the force. The main cracks that revealed the characteristics of the paint layer in the original works were similarly produced in counterfeit works. Although the result was not exactly the same as the original crack, the thickness of the painted layers and the composition of the medium were manipulated to produce counterfeit cracks similar to naturally occurring cracks.

In this study, 34 works were rented, and only three of them were selected as representative works. All these works were painted in oil paint on canvas frames. Some works were created for more than 10 years ago, but most of them were created within the past five years. Cracks occurred in eight artworks and the representative causes of cracks are drying or aging. The OCT cross-sectional images of cracks in five artworks in which cracks occurred were obtained and the geometrical parameters of the cracks were quantified **(Figs S2-S11 in S1 File)**. These results were referred to analyzing the cracks of three representative works in this main text.

## 3. Results

### 3.1 Appearance and location of cracks selected in *Artwork 1* and *Artwork 2* to evaluate cracks induced by drying of the paint layers using the OCT system

The target regions for OCT analysis were selected at three locations where cracks were generated in each work. The three selected points represented crack locations that were expected to provide particularly meaningful information for art authentication based on the OCT analysis of cracks.

#### Artwork 1

**Fig 3:**
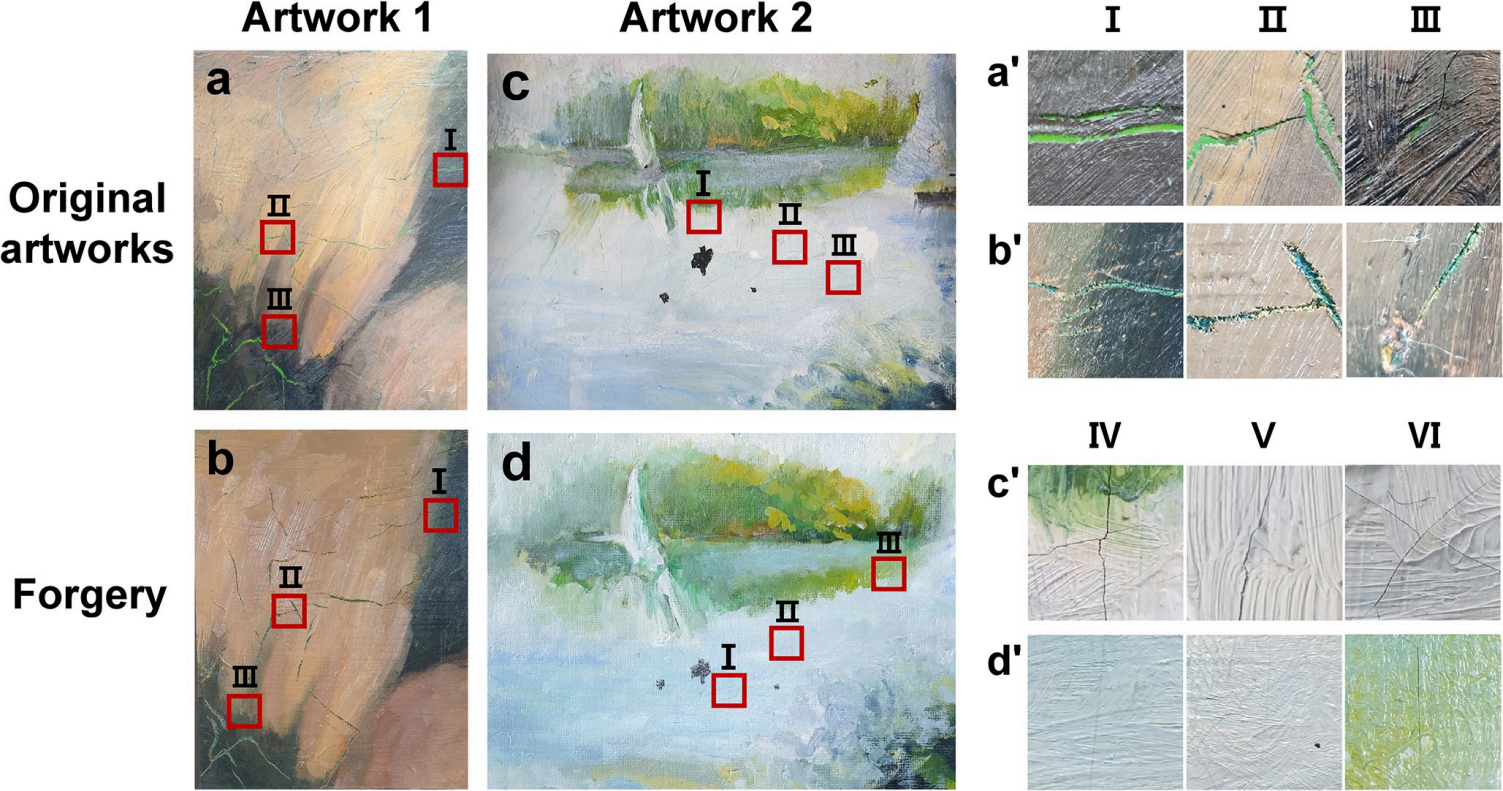
Images of *Artwork 1* **(a, b)** and *Artwork 2* **(c, d)** showing the area selected by the OCT system and enlarged views of the cracks in each area. The cracks were selected because the paint characteristics of each work were prominent. They were then analyzed with the OCT system. Enlarged images of original cracks **(a’, b’)** and fake cracks **(c’, d’)** at the selected location in *Artwork 1 and Artwork 2*.

**Fig 3A** shows the varnish-processed oil painting with light brown and darker brown upper layers on top of a light green undercoat layer applied to a canvas. Cracks occurred in both the light brown and darker brown upper layers, except for the under layer of paint. The cracks occurred because the proportion of oil painting media used in the upper paint layers (light brown/dark brown) was not adequately controlled. Forged cracks were produced in both the light and dark brown paint layers to match the locations at which the original cracks were generated. The cracks were produced by drawing thin lines similar to cracks produced using sharp knives on the paint layers with detailed control so that the color on the bottom side of the paint layer was visible **(Fig 3B)**.

#### Artwork 2

In **Fig 3C**, the varnish-processed artwork has multiple layers composed of a black underpainting layer and upper layers with a mixture of white, light blue, and green colors. Cracks occurred on the upper layers because the upper painted layers contained a relatively small amount of medium. The under layer of the artwork was painted black, and the upper layer was composed of a mixture of white, light blue, and light green paints. Unlike *Artwork 1*, cracks were generated because there was only a small amount paint medium in both the upper and under layers. Some cracks were relatively thin; therefore, the color of the underlayer was not visible. Although the location of the cracks in the forged work was not similar to that of the original cracks, the forged cracks were generated so that it was difficult to distinguish them from original cracks with the naked eye **(Fig 3D)**. In addition, the thickness of the forged cracks was controlled to be as similar as possible to that of the original cracks.

### 3.2 Quantitative analysis of the original cracks and forged cracks to explain drying cracks of the paint layers on *Artwork 1* and *Artwork 2*

**Fig 4:**
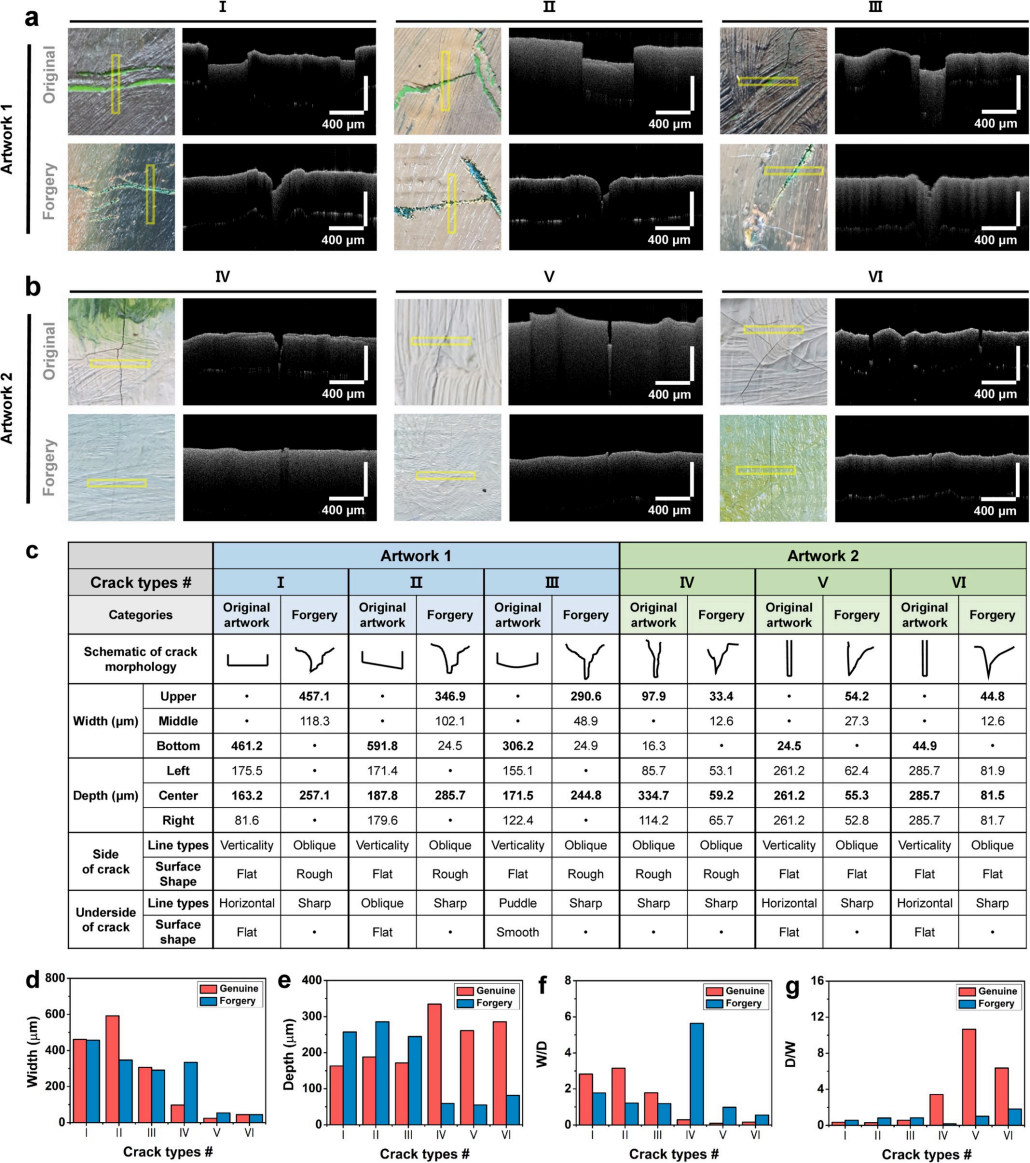
Comparison between the cross-sectional characteristics obtained from the cracks in the original artworks and the counterfeit cracks using the OCT system. **(a)** Cracks in *Artwork 1* caused by the small amount of medium in the upper layer but formed with a wide rectangular shape. The forged cracks in *Artwork 1* appear similar in depth to the original cracks, but unlike the original cracks, which are rectangular, they have an inverted triangle shape. **(b)** Original cracks in *Artwork 2* caused by the small amount of medium in the under layer. The cracks were formed in a thin and very deep rectangular shape. Unlike the original cracks, the forged cracks in *Artwork 2* are very shallow in depth. **(c)** Quantitative analysis of the OCT cross-section images of the original and forged cracks of *Artwork 1* and *Artwork 2*. The representative values of the dimensions of the original and forged cracks selected for analysis, were compared by **(d)** width, **(e)** depth, **(f)** width/depth, and **(g)** depth/width.

The cracks created in the original and counterfeit artworks were measured using the OCT system **(Fig 4A)**. The cross-sectional images of the cracks were compared to analyze the differences in the morphologies of the original and forged cracks, such as the depth and shape **(Fig 4B)**. These images contain adequate information because they can identify the 3D visual features of the crack that cannot be observed by the naked eye or a microscope. The widths (top, center and bottom widths) and depths (left, center, and right depths) of each crack were quantified using cross-sectional image analysis. **Fig 4C** shows the morphology of the cracks formed in the forged and original artworks. The quantitatively measured values of the scale parameters of each crack were classified into various types. These representative values were selected from the largest values of the crack widths and depths and were used to compare the original and counterfeit artworks **(Fig 4D–4G)**.

#### Artwork 1 (Fig 4A)

In **Fig 4**, cracks caused by the combination of media occur for each color, but the shape of the cracks for each color was not significantly different. The most prominent feature in the OCT cross-sectional image of the original cracks in *Artwork 1* is a rectangular shape in which the side interface of the crack is shaped like a cliff and the bottom surface of the cracks is flat. This indicates that, when the media in the upper painted layer dried, several gaps were left in the paint, resulting in a complete loss of internal adhesion. As the upper layer dried, the internal bonding decreased, and hence, the side of the crack was clean. However, the surfaces of the forged cracks tended to be uneven in terms of surface roughness. The top sides appeared similar, but there was an evident difference in the cross-sectional OCT images.

The cracks of the original artworks had a rectangular shape; therefore, each parameter of the crack width showed similar values. On the other hand, the cracks in the forged artworks had inverted triangular shapes, the measured values were different for each parameter type, and the bottom parameter values were not measured. The depth of the cracks in the counterfeit and original artworks differed by (Ⅰ) 93.9 μm, (Ⅱ) 97.9 μm, and (Ⅲ) 73.3 μm **(Fig 4C)**. It appears that the depth of the crack increased as a force was applied to the painting layer with a knife to create a width similar to that of the original cracks.

#### Artwork 2 (Fig 4B)

The under layer of paint had a larger amount of medium than the upper layer. As the medium solutions dried, small empty pores were created inside the under painted layer. Consequently, the adhesion inside the under painted layer decreased and this phenomenon appears to have affected the occurrence of cracks in the upper layer of the paint. The main characteristic of the cracks is that the width of the cracks is very small; thus, the color of the under layer is not visible, and the aspect ratio (depth/width) of the cracks is large. The original cracks had thin and deep rectangular shapes, whereas the forged cracks had triangular shapes with rough surfaces. A concentrated force was applied with knives to create forged cracks with a width similar to that of the original cracks. Consequently, the forged cracks appeared to be relatively shallow.

**Fig 5:**
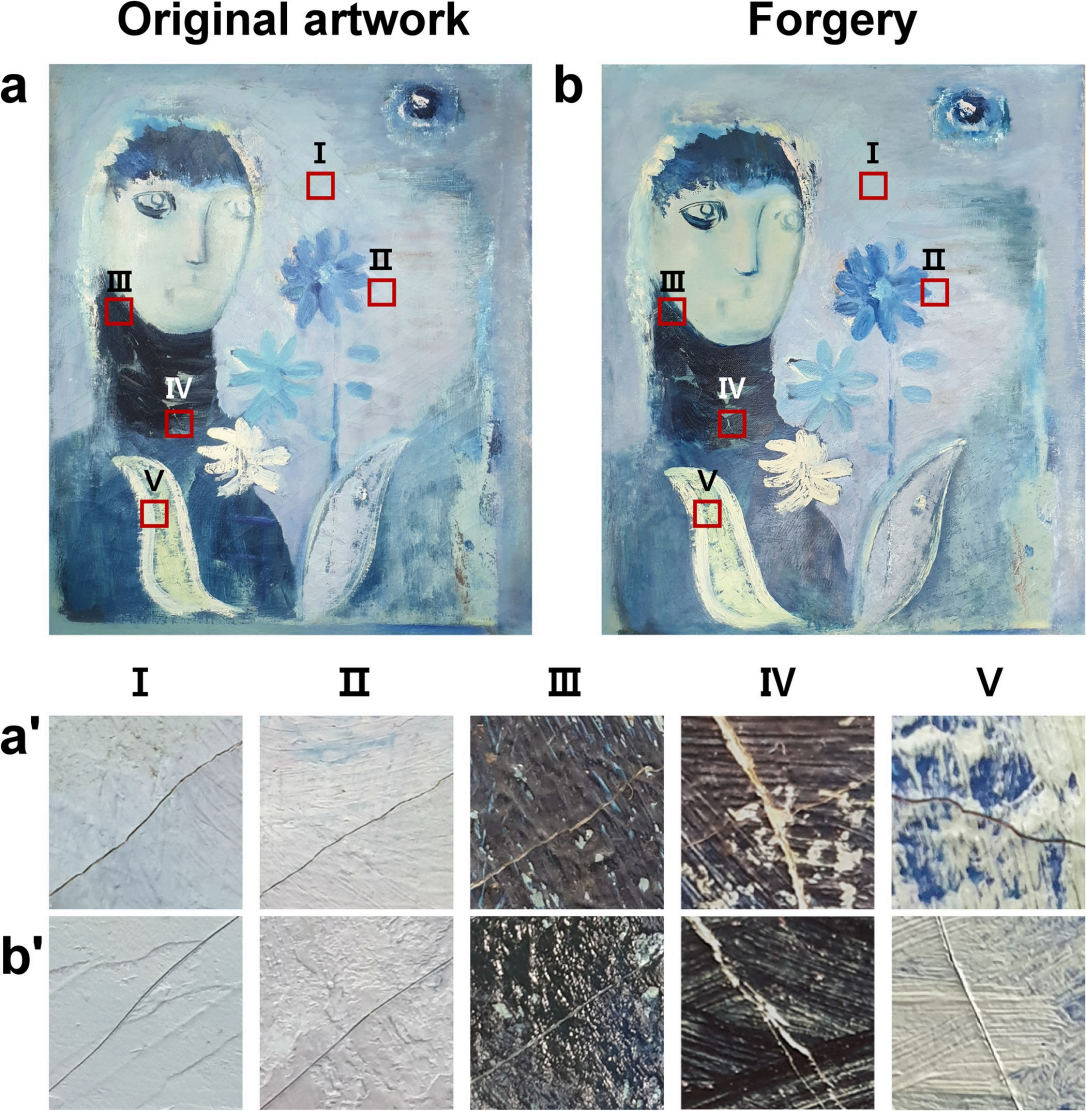
Images showing the location of cracks in **(a)** the original and **(b)** forged oil paintings. The enlarged images of cracks selected as the OCT measurement area in **(a’)** the original artwork and **(b’)** the forged artwork. In various areas of this artwork, cracks were created by **(Ⅰ, Ⅱ)** canvas aging, **(Ⅲ, Ⅴ)** paint layer drying, and **(Ⅳ)** physical impact on the layer. The forged cracks were created considering the characteristics and causes of the original cracks.

A medium combination problem occurs in the under layer of paint. The depth was observed to be approximately 2.5 times larger than the width of the crack **(Fig 5B)**. In the case of thin and deep cracks, the cross-sectional shape of the crack was either thin rectangular or inverted triangular. The weakened bonding force in the under layer of paint affected the upper layer, and it was judged that the crack at the upper part had small width.

### 3.3 Location and enlarged images for a comparison of original and forged cracks in *Artwork 3* to explain cracks induced by drying, canvas aging, and direct physical impacts of the paint layers

We borrowed oil paintings on canvas created by Joonja Jun to analyze the original cracks generated by other factors and the forged cracks. The five cracks were classified into five types according to the characteristics of the original artworks and the cause of crack occurrence.

#### Artwork 3

The artist attempted to overlay the surface of the artwork with light purple paint to cover naturally occurring cracks **(Fig 3E)**. This work was selected for art authentication based on OCT analysis because of the occurrence of various types of cracks in the paint layers with varied thicknesses. As the new canvas has good elasticity and does not crack easily, a canvas that was produced approximately 25 years ago was used as the substrate for the forged artwork. In addition, the thickness of the paint layer on the forged artwork was similarly formed by observing the original product with the naked eye **(Fig 3F)**.

#### Craquelure Ⅰ

**Fig 6:**
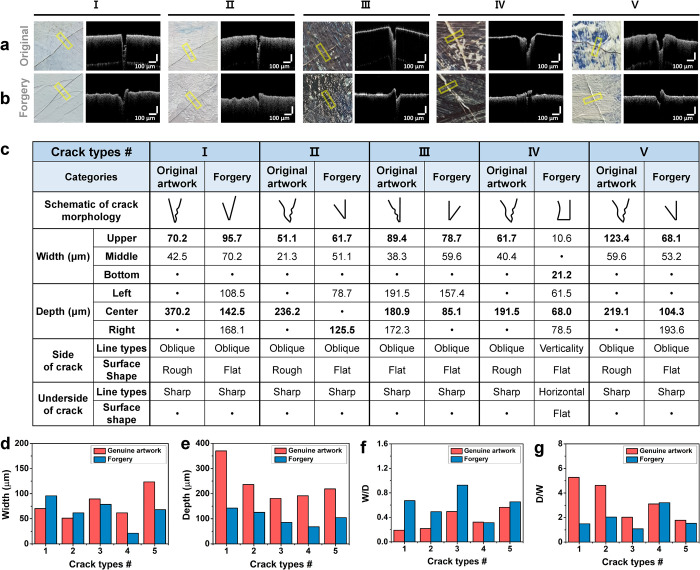
Comparison between the OCT cross-sectional images of (a) the original and (b) forged artworks for art authentication. (c) Quantitative analysis of the OCT cross-section images of the original and forged cracks of *Artwork 3*. The representative values of the dimensions of the original and forged cracks selected for analysis were compared by (d) width, (e) depth, (f) width/depth, and (g) depth/width. (Ⅰ) Cracks caused by the aging canvas made in areas covered in a thin layer of paint. (Ⅱ) Cracks caused by the aging canvas made in areas where the paint layer was relatively thicker. (Ⅲ) Cracks caused by the drying paint layer and made in areas with a thin layer. (Ⅳ) Cracks made by applying a physical impact to the paint layer. (Ⅴ) Cracks caused by the drying of the paint layer and made in area with a thick layer. The side paint layer of the forged cracks protrudes and has a much shallower inverted triangle shape than the original cracks.

These cracked areas occurred owing to the aging and shrinkage of the canvas at the paint layers that were thinner than the others **(Fig 6A’ Ⅰ)**. The speculative reason is that wrinkles appear in the same direction on the back of the canvas as the cracks advanced on the painted surface. These cracks occurred at locations painted with various pigments, regardless of the paint layer colors. To imitate the visual morphology of the original crack, thin mass tools were used to perform finely tuned slashing to create long paths of dynamic cracks in the painting layer of the forged work **(Fig 6B’ Ⅰ)**. Consequently, forged cracks with thicknesses and directions similar to those of the original cracks were created.

#### Craquelure Ⅱ

Similar to the reason for the occurrence of *Craquelure I*, *Craquelure Ⅱ* also occurred in the aged area of the canvas **(Fig 6A’ Ⅱ)**. Cracks in the paint layers commonly occur in the direction of the thick wrinkles on the back of the canvas, but the difference is that the paint layer at this location *(Craquelure Ⅱ)* is relatively thicker. Forged *Craquelure Ⅱ* was produced using the same slashing method used to create forged *Craquelure I* **(Fig 6B’ Ⅱ)**.

#### Craquelure Ⅲ

*Craquelure Ⅲ* may appear on painted surfaces during the drying process owing to chemical environmental changes in the layers of paint **(Fig 6A’ Ⅲ)**. Cracks were generated across painted layers with a mixture of three colors that were applied with a thickness greater than that of the surrounding layers of paint. In the middle of the paint layer, the painting mediums were poorly mixed, which led to drying cracks occurring in large areas of the painted surface. Forged cracks were also produced in a pale-yellow color of the undercoated layer through a deliberate process in which the force was appropriately adjusted with a thin knife **(Fig 6B’ Ⅲ)**.

#### Craquelure Ⅳ

*Craquelure Ⅳ* occurred owing to unintentional physical impacts on the completed painting by the artist and appeared to be scratched by an ambiguous tool **(Fig 6A’ Ⅳ)**. The ivory color of the under layer of paint was visible in the exposed area. During the drying process, the cracks widened with the formation of several branches. When these cracks were forged, they were created by applying deliberate force to the painted surface using a stainless-steel tool. The width of the forged crack was suitably widened, and the under surface layer was clearly visible, similar to the original cracks **(Fig 6B’ Ⅳ)**.

#### Craquelure Ⅴ

Similar to the reason for the occurrence of *Craquelure Ⅲ*, *Craquelure Ⅴ* occurred in the thickest areas of the artwork **(Fig 6A’ Ⅴ)**. Cracks were observed across both the upper layer (white) and the sublayer (blue). To replicate the original cracks, a thicker knife was used to apply a relatively stronger force to the painted surface layer **(Fig 6B’ Ⅴ)**.

### 3.4 Quantitative analysis of the original and counterfeit cracks to explain the cracks caused by drying, canvas aging, and direct physical impacts of the paint layers on *Artwork 3*

#### Craquelure Ⅰ

The cross-section of the original *Craquelure Ⅰ* had an inverted triangular shape in the upper painted layer **(Fig 6A Ⅰ)**. The shape became a sharp point toward the undercoated layers, and the height of both side layers along the crack remained approximately constant. The upper painted layers were affected by shrinkage that occurred owing to the aging of the canvas, and the cracking process may have advanced in the top layer of the paint. Thin thread-like structures were exposed on the inner sides of the cracks. This may have occurred because the ratio of the medium mixture to oil painting was different for each colored layer, which resulted in incomplete separation inside the painted layers. Similarly, the cross-sectional morphology of the forged cracks was a similar inverted triangle, but these cracks had a larger gap and a slightly shallower depth **(Fig 6B Ⅰ)**. In the forged cracks, one side of the painted layers had a protruded outer surface because of the physical influence of drawing of the crack lines.

The natural cracks were generated by canvas shrinkage and were 70.2 μm in width and 370.2 μm in depth **(Fig 6C Ⅰ)**. In contrast, the forged cracks were approximately 95.7 μm in width and 142.5 μm in depth. This observation may indicate that forged cracks are wider and shallower than the original cracks. There is a statistically significant difference of approximately 227.7 μm in the crack depth between the original and forged cracks. Although the force was finely adjusted to create a small width, it was almost impossible to control the depth with precision.

#### Craquelure Ⅱ

The original cracks had an inverted triangle with a slight incline **(Fig 6A Ⅱ)**. The width of the cracks was narrower than that of the original *Craquelure Ⅰ*. The inner spacing of the cracks had an uneven structure without thread-like fibers. In the cross-sectional image of the forged cracks, one side of the painted layers had a slightly overhung outer painted surface **(Fig 6B Ⅱ)**. The forged cracks had a relatively shallow depth, and the sidewalls of the cracks were similar to those of the forged *Craquelure Ⅰ*.

The forged *Craquelure Ⅱ* were also wider and shallower than the original cracks, as in the case of the forged *Craquelure Ⅰ*. When compared with than *Craquelure Ⅰ*, the crack widths were relatively smaller by 20 μm, but the depth was shallower by 134 μm **(Fig 6C Ⅱ)**. The original *Craquelure Ⅰ* and *Ⅱ* were created by canvas aging and *Craquelure II* occurred in thicker paint layers than did in *Craquelure Ⅰ*. However, it is difficult to forge cracks based on the tendency of the natural crack shapes produced according to the thickness of the paint layer. Consequently, the shapes and dimensions of the original *Craquelure Ⅰ* and *Ⅱ* were different, but the forged *Craquelure Ⅰ* and *Ⅱ* had similar shapes, widths, and depths.

#### Craquelure Ⅲ

The cross-section of the crack had an inverted triangle shape with a tip having a relatively blunt shape **(Fig 6A Ⅲ)**. The central axis of the inverted triangle was slightly inclined. The small width of the forged cracks was produced to reveal the color of the underlying paint layer, similar to the original cracks **(Fig 6B Ⅲ)**. Although the widths of the two cracks appeared similar, the main difference between these two cracks could be accurately distinguished according to the depth and shape from the OCT cross-sectional images.

The aging conditions of the canvas directly contributed to the occurrence of *Craquelure Ⅰ* and *Ⅱ*, but *Craquelure Ⅲ* occurred as a direct result of the drying of the upper paint layers. The width of the crack was approximately 89.4 μm, which is relatively large. Conversely, the depth was relatively shallow at 180.9 μm **(Fig 6C Ⅲ)**. *Craquelure Ⅰ* and *Ⅱ* were produced by the influence of shrinkage in the canvas below the paint layer and were relatively thinner and deeper. In contrast, *Craquelure Ⅲ* was caused by the drying of the paint layer and was represented a reverse shape with a relatively large width and shallow depth.

#### Craquelure Ⅳ

Cracks with a large width are visible on the of the outer painted layer, and a sharply thin inverted triangle shape with a sharp tip is evident in the deeper undercoated layer **(Fig 6A Ⅳ)**. The forging process was conducted under condition in which the coating thickness was unknown. These forged cracks were produced to reveal the ivory-color of the under layer similar to the original cracks, but the difference in depth and shape are evident in the OCT cross-sectional image **(Fig 6B Ⅳ)**.

The original cracks had a larger upper width in the shape of an inverted triangle, and the forged cracks showed a large bottom width. The original crack was approximately 191.5 μm in depth, which is greater than the depth of the forged crack of 68.0 μm **(Fig 6C Ⅳ)**. This may indicate that the original cracks were initially scratched, and after a considerable amount of time, the cracks deformed in width and depth under paint conditions such as paint drying. In addition, the width/depth ratio of the original and forged cracks were similar.

#### Craquelure Ⅴ

The sidewall of the cracks appeared to be relatively neat, as the painted layer appeared to have dried the most **(Fig 6A Ⅴ)**. The original *Craquelure Ⅴ* had a large width, but the color of the under layer was not visible. As the paint layer was thick, the crack was not sufficiently deep to reveal the color of the under layer. The forged *Craquelure Ⅴ* was extended and widened by applying a larger force than that used of producing the other forged cracks **(Fig 6B Ⅴ)**. The sidewall of the cracks appeared to be relatively neat, as the painted layer appears to have dried the most. Therefore, the upper layers were extruded from crack edges. In addition, the side of the forged crack was neatly formed in a straight line, but the detailed appearance with a zigzag shape from the paint layer of the original crack could not be forged.

*Craquelure Ⅴ* was 123.4 μm in width and 219.1 μm in depth **(Fig 6C Ⅴ)**. Compared with *Craquelure Ⅲ*, which occurred in the thinner paint layer, *Craquelure Ⅴ* was 34.0 μm wider and was 38.2 μm deeper. This was observed in the contrast to the original *Craquelure Ⅰ* and *Ⅱ*, which were affected by the canvas underneath the painted layers. If natural cracks are directly influenced by the condition inside the upper painted layers, thicker painted layers generate cracks with a greater width and depth at the painted surface. In contrast, if cracks occur because of the influence of the canvas layer, the shape of cracks become thinner and shallower. The forged cracks were 68.1 μm in width and 104.3 μm in depth. Various cracks with similar widths can be artificially produced, but the depth is difficult to control. These cracks are not as deep as the original cracks **(Fig 6C–6G)**.

## 4. Discussion

Cracks in works of art occur for three reasons: the drying of the paint layer, factors related to canvas aging, and direct physical impacts on the painted layer. First, cracks occurred in all three works owing to the drying of the painted layers. In *Artwork 1*, wide cracks occurred in the upper layers, which contained a larger amount of the medium than the under layer. However, thin cracks indirectly occurred in the upper layers of *Artwork 2* because the under layers had a larger amount of medium than the upper layers. As drying advanced, the medium inside the painted layers evaporated, leaving several empty spaces in the painted layers. The adhesion between the painted layers decreased, resulting in cracking. If this cracking occurs mainly in the upper layer, there is a tendency for the cracks to widen, as in *Artwork 1* **(Fig 4A)**. The entire thickness of the upper layer was split, and the depth of the crack corresponded to the thickness of the upper layers. Conversely, if cracking occurs mainly in the under layers, the reduced adhesion in the under layers affects the upper layers. Thin and deep rectangular cracks were observed in the upper layers of *Artwork 2* **(Fig 4B)**. In *Artwork 3*, the paint layer dried over a long period of time in addition to the aging of the canvas. Unlike *Artwork 1* and *2*, cracks occurred owing to factors related to both the upper and under layers. Unlike for the previous cracks, the cross-sectional shape of an inverted triangle was observed. When the paint layer was thicker, cracks were wider and deeper.

The second factor was the aging of the canvas, which affected the paint layers and generated cracks. These cracks were observed only in *Artwork 3* and occurred in both the thin and thick areas of the artwork. When the paint layers were thick, the width of the cracks became thinner and deeper. The cause of cracking appeared in the support under the paint layers. A weakened adhesive force is applied in the under layer so that the upper layers have cracks, and the thinner the upper paint layers, the easier it is to produce cracks. Therefore, in this case, larger values were observed for crack width and depth.

Third, cracks occurred owing to physical shock and were observed only in *Artwork 3*. A crack had been created in the original work owing to an unintended physical impact, but a long time had passed since then. The cracks in the forgery were also created by physical impact, but the difference from the original cracks was the length time elapsed. The much older crack was observed to be wider and deeper. The analysis of these two cracks reveals how deformation occurs over time in the crack generated by physical impact. Based on the above aspects, it is possible to verify whether a crack has been forged or whether the time elapsed since the occurrence of the crack is appropriate compared with the time of production of the work for authenticity assessment. According to the results of the analysis, the width and depth ratios of cracks in the same artwork differ according to the cause of the crack occurrence; therefore, it is necessary to measure several cracks and compare the width and depth ratios **(Fig 6B–6D)**.

## 5. Conclusion

OCT technology has been proven to have the potential to analyze the characteristics of natural cracks and simultaneously identify differences from forged cracks. The differences between natural and forged cracks were analyzed by quantitatively measuring the parameters (width, depth, and cross-sectional shape) of the cracks. In this study, the characteristics of cracks in works that can be measured are summarized. Using artificial and natural methods, it was confirmed that the crack widths were similar. The results inferred that it is not sufficient to determine the authenticity of artwork with only one factor i.e., crack width and that the depth of cracks can provide a useful basis for determining the authenticity of an artwork. Natural cracks can become deep as the thickness of the upper paint layer in rectangle or inverted triangle shape, but artificial cracks are not formed in the same way as these natural cracks. The depth of artificial cracks is not as finely controlled as that of natural cracks. As there is a limit to controlling the depth and width of natural cracks, it is possible to analyze whether the cracks are forged. If the cross-sectional shape is considered in addition to the depth, the difference between natural and artificial cracks is revealed, which can further increase the possibility of the successful detection of authenticity. The cross-section of natural cracks can have various shapes, such as a rectangle, trapezoid, inverted triangle with a clean surface, inverted triangle with an internal microstructure, and inverted triangle with a large width only in the upper layer. However, most artificial cracks have a similar inverted triangle shape and two dimensions; wide/deep or narrow/shallow. The distinct difference between natural and artificial cracks according to the crack parameters makes OCT technology useful for appraising the authenticity of artworks. The OCT results presented in this study are limited in that only artificial cracks made by knives were compared with natural cracks. If cracks made by other factors, such as crackle paste, are evaluated using OCT, the 3D images and geometrical properties of the cracks will be obtained without technical issues; however, sufficient information for the discrimination of cracks in artworks can be unclear to obtain. Therefore, combining the foundation of this study with further studies to analyze other cracks is necessary to establish a more specialized OCT system to understand crack properties better for art authentication. Furthermore, by continuously measuring the surface of an artworks using OCT technology and observing the development of cracks and changes in the surface, various types of information to prevent forgery of the artwork can be collected. This study demonstrated that the difference between natural and artificial cracks in oil paintings can be analyzed using OCT technology, which has shown breakthrough potential for determining the authenticity of artworks.

## Supporting information

S1 File It contains all the supporting figures with captions. (PDF)
