# Correction: Investigation of craquelure patterns in oil paintings using precise 3D morphological analysis for art authentication

**DOI:** 10.1371/journal.pone.0293153

**Published:** 2023-10-12

**Authors:** Soojung Kim, Sang Min Park, Seongjin Bak, Gyeong Hun Kim, Chang-Seok Kim, Joonja Jun, Chang Eun Kim, Kyujung Kim

There are errors in the Funding section. The correct Funding statement is: This work was supported by the National Research Foundation of Korea (NRF) grant funded by the Korea government (MSIT) (No. 2020R1A2C4002732, 2021R1A4A2001827) and Korea Medical Device Development Fund (KMDF_PR_20200901_0103). The funders had no role in study design, data collection and analysis, decision to publish, or preparation of the manuscript.
