# Supplementary material for: Investigation of craquelure patterns in oil paintings using precise 3D morphological analysis for art authentication
Source: PLoS One. 2022 Jul 28;17(7):e0272078. doi: 10.1371/journal.pone.0272078 (PMC9333328; doi:10.1371/journal.pone.0272078)
Supplement: S1 File — (PDF) [file pone.0272078.s001.pdf]

Investigation of craquelure patterns in oil paintings using precise 3D morphological analysis for art authentication

Soojung Kim[1], Sang Min Park[1], Seongjin Bak[1], Gyeong Hun Kim[1], Chang-Seok Kim[1], Joonja Jun[2], Chang Eun Kim[2], Kyujung Kim[1,3,*]

[1]*Department of Cogno-Mechatronics Engineering, Pusan National University, Busan, 46241, Republic of Korea*

[2]*Department of Fine Arts, Pusan National University, Busan, Republic of Korea*

[3]*Department of Optics and Mechatronics Engineering, Pusan National University, Busan, 46241, Republic of Korea*

**Author e-mail address: k.kim@pusan.ac.kr*

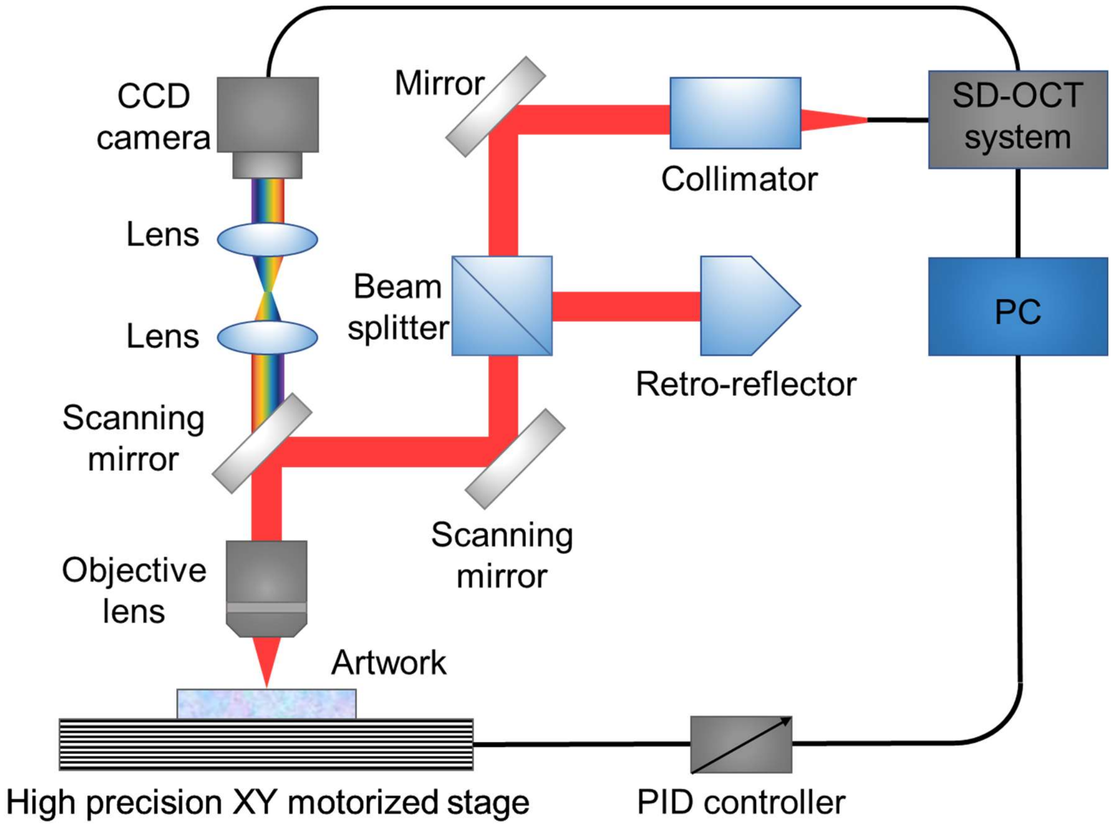

Figure S1. Schematic of the OCT system-based the Michelson Interferometer. OCT system prepared to measure the crack area of artwork. This system can stably fix artworks on the stage and move them in units of at least 10 μm, along the XY axis and acquire 3D data of art objects.

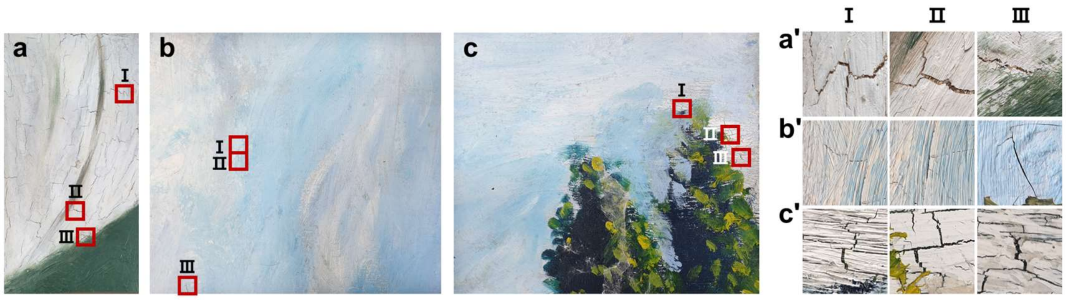

Figure S2. Enlarged images of original cracks at the selected location in (a’) ***Artwork S1, (b’) Artwork S2 and (c’) Artwork S3***. These works were painted as oil paintings on canvas, and the works were not fixed to a wooden frame and were kept partially cut out. These are the works of the artist Eunyoung Ko.

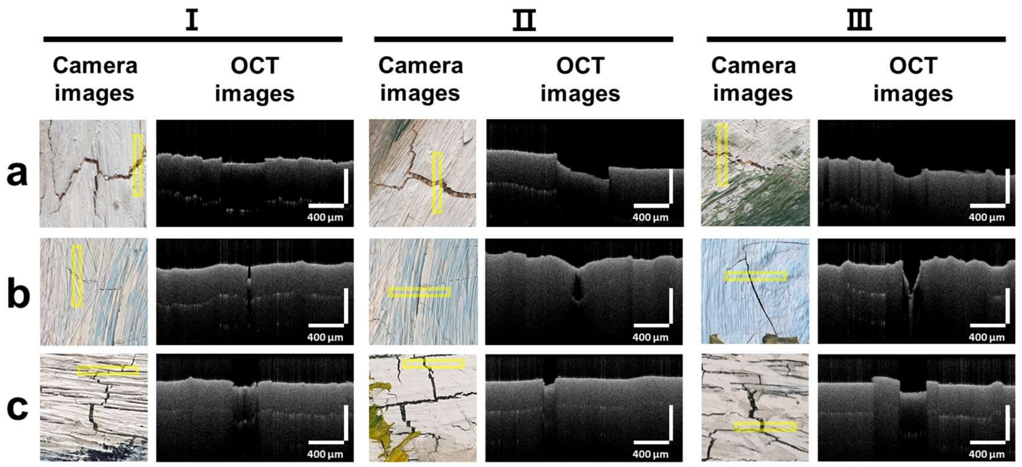

Figure S3. Enlarged images of original cracks at the selected location in (a) *Artworks S1*, (b) *Artwork S2*, and (c) *Artwork S3*. (a) Cracks occurring only in the white color paint layer because the medium amount for each color was not properly adjusted. There were no cracks in the green paint layer. (b) Cracks occurring in places where the amount of paint is large, and the paint layer is quite thick. (c) Cracks caused by the force being pulled on the canvas because the canvas is not properly fixed to the frame.

Crack samples		A			B			C		
Crack types #		I	II	III	I	II	III	I	II	III
Schematic of crack morphology		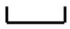	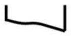	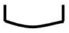	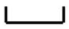	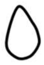	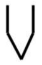	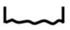	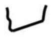	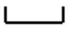
Width (μm)	Upper	•	**583.7**	•	**16.3**	•	**195.9**	•	**142.8**	•
	Middle	•	•	**306.1**	•	**130.5**	126.5	•	102	•
	Bottom	**484.2**	•	•	36.7	•	•	**212.2**	•	**302**
Depth (μm)	Left	81.6	65.3	65.3	142.9	•	•	73.4	69.4	110.2
	Center	**69.4**	**151**	**122.4**	**142.9**	**371.4**	**383.7**	**65.3**	**82.6**	**130.6**
	Right	40.8	147.3	53	142.9	•	•	44.9	57.1	110.2
Side of crack	Line types	Verticality	Verticality	Verticality	Verticality	Oblique	Oblique	Verticality	Oblique	Verticality
	Surface Shape	Flat	Flat	Flat	Flat	Smooth	Smooth	Flat	Rough	Flat
Underside of crack	Line types	Horizontal	Oblique	Puddle	Horizontal	Puddle	Sharp	Horizontal	Horizontal	Puddle
	Surface shape	Flat	Rough	Smooth	Flat	Rough	•	Rough	Flat	Smooth

Figure S4. The cross-sectional shape, width, and depth of crack in (A) *Artwork S1*, and (B) *Artwork S2*, and (C) *Artwork S3*. The shape of the crack side, and the shape of the underside of the crack were described. (A) The crack A was observed roughly and the surface of the underside of the crack was not clean. (B) The crack B appeared to have a thin crack on the outside, but a wider crack could be observed inside. (C) The width of the crack was larger than the depth.

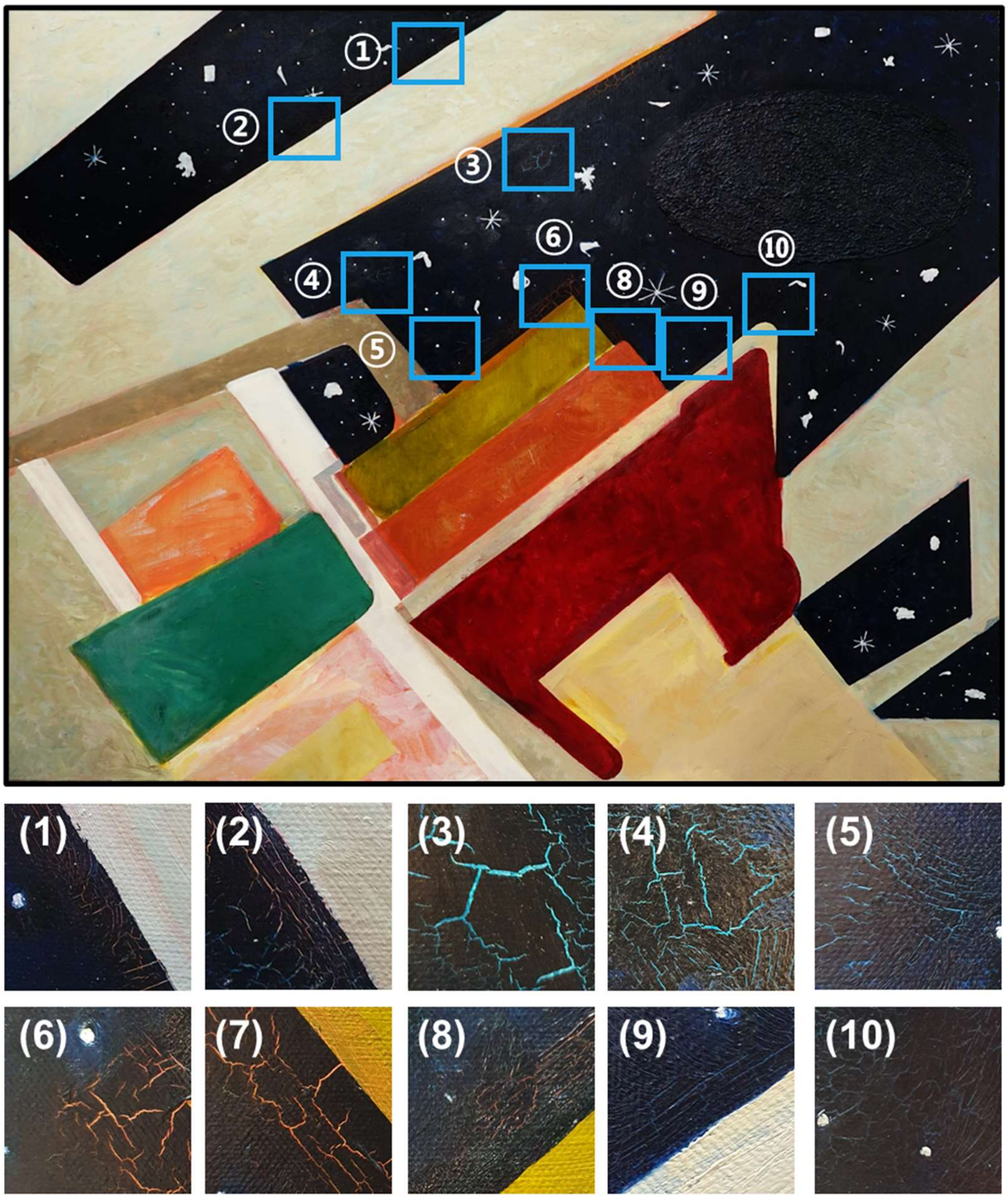

Figure S5. The title of Artwork S4 is '*A man who stares at space*' (116.8 cm × 91.0 cm), produced by Dongyeon Kim, and it is a work painted with oil painting. Cracks occurred only in the blue paint layer, and the thickness of the cracks varies. The base color of the cracked area is light blue or orange. There were relatively many cracks in the orange base layer. Ten locations were selected from the cracked areas and analyzed by OCT system.

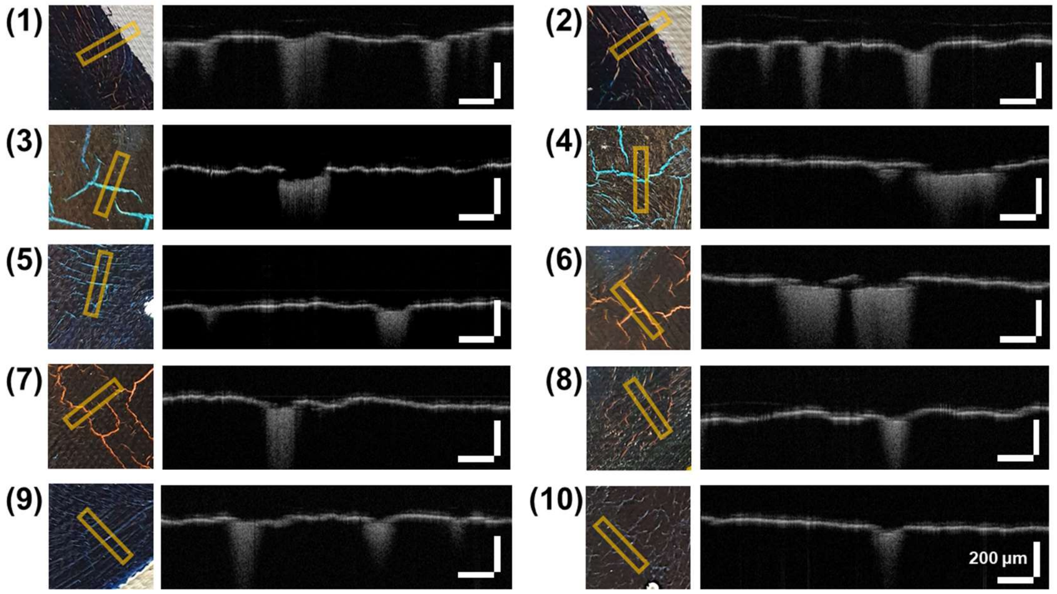

Figure S6. In Artwork S4, cracks in various areas were analyzed with an OCT system and cross-sectional images were acquired to see how the cross-sectional shape of the crack generated in the same color changes. Various cracks were measured, such as cracks showing the underlying color and cracks thin enough to be invisible. In addition, cracks showing a combination of various types with surrounding cracks such as straight-line cracks, grid pattern cracks, and tangled cracks were measured.

Crack types #		1	2	3	4	5	6	7	8	9	10
Schematic of crack morphology		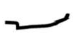	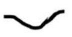	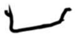	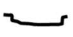	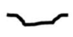	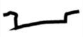	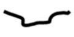	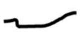	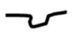	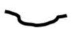
Width (μm)	Upper	177.8	201.9	223.1	342.9	172.6	270.4	126.2	141.3	•	128.5
	Middle	•	•	200.6	326.3	128.5	197.7	97.1	•	•	•
	Bottom	102.4	51.7	133.5	301.5	92.8	167.9	80.5	99.2	24.5	61.9
Depth (μm)	Left	10.71	•	59.9	31.0	•	59.5	35.5	28.6	23.9	19.2
	Center	17.86	48.8	54.8	46.4	33.3	53.57	26.19	23.8	21.4	28.1
	Right	•	•	52.4	11.9	•	25.0	28.57	15.5	16.7	16.7
Side of crack	Line types	Oblique	Oblique	Verticality	Verticality	Oblique	Verticality	Verticality	Oblique	Oblique	Oblique
	Surface Shape	Flat	Flat	Rough	Flat	Flat	Flat	Flat	Flat	Flat	Flat
Underside of crack	Line types	Puddle	Puddle	Horizontal	Horizontal	Horizontal	Horizontal	Horizontal	Puddle	Puddle	Puddle
	Surface shape	Smooth	Smooth	Flat	Flat	Flat	Flat	Flat	Smooth	Smooth	Smooth

Figure S7. Thin cracks generally have a smooth curved shape, but thick cracks have a rectangular shape. It seems that the cross-sectional shape of cracks that can occur due to the thin thickness of the paint layer is limited. In addition, the information on the side of the crack was limited in the form of a smooth curve or a straight line. It tends to appear in a smooth curved shape rather than a sharp triangular-shaped crack cross section.

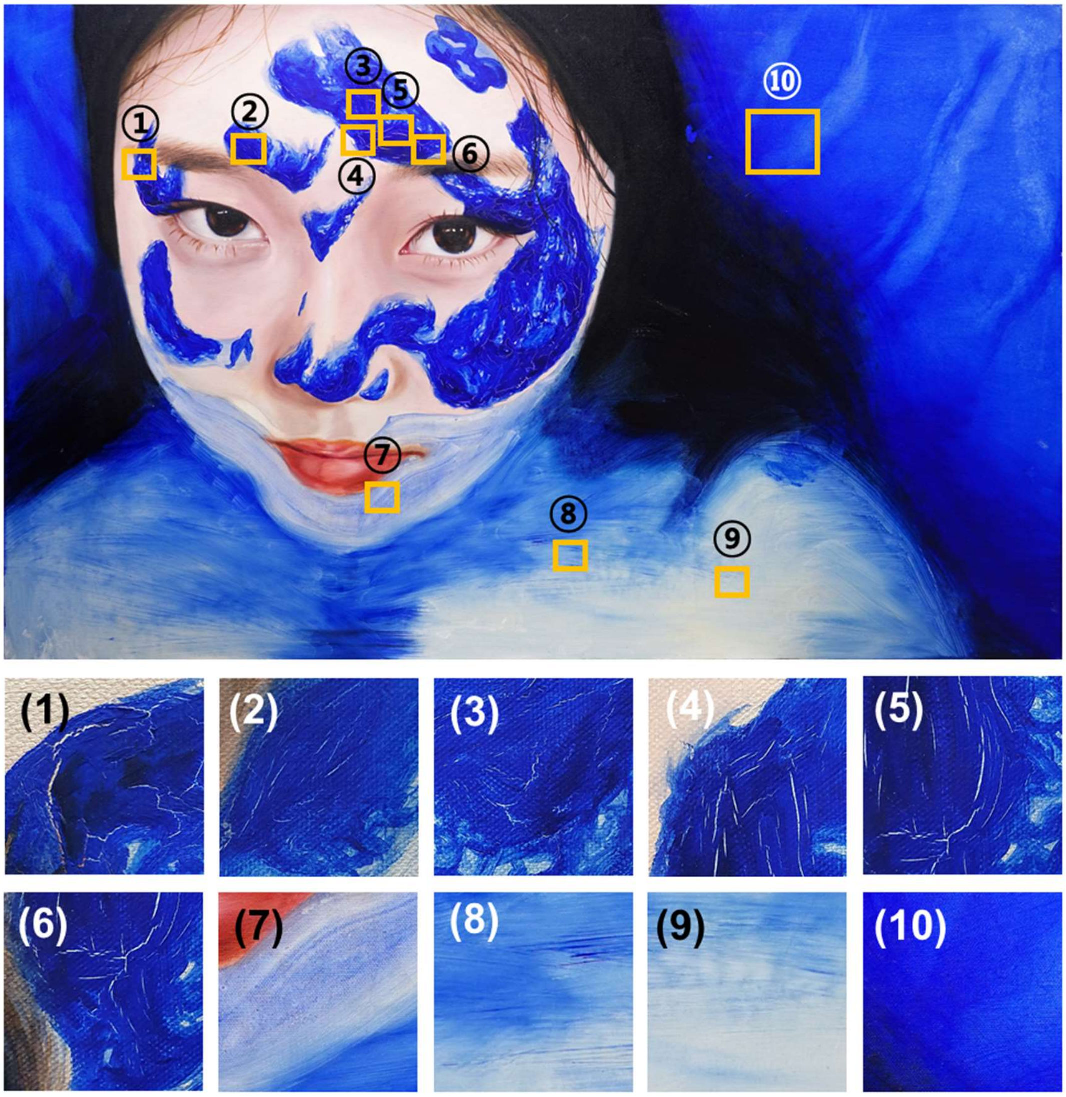

Figure S8. The title of Artwork S5 is '*Self-portrait*' (116.8 cm × 72.7 cm), produced by Kangmi Ha, and it is an oil painting on canvas. In the work, many cracks occurred only in the blue paint layer overlaid with another color. There were no cracks in the background blue paint layer painted directly on the canvas. Even in the same color, the area without cracks was also analyzed with the OCT system to find out what difference appeared in the background and the paint of the person. Most cracks tend to appear like long threads.

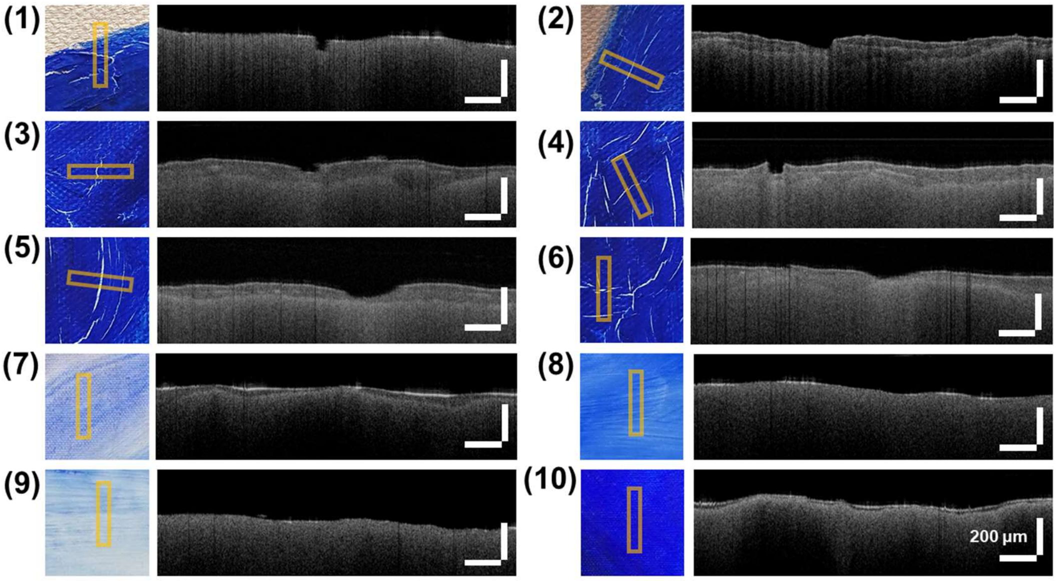

Figure S9. (1) to (6) are the cracked areas, (7) to (10) are the cracked areas, and cross-sectional images according to the presence of cracks were compared. The cross-sectional shape of the thick crack has a smooth curve shape and shows a shallow depth, whereas a distinct rectangular shape was observed in the shallow crack.

Crack types #		1	2	3	4	5	6
Schematic of crack morphology		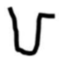	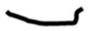	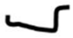	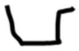	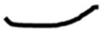	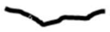
Width (µm)	Upper	67.1	189.7	101.1	89.8	295.3	296.3
	Middle	35.1	133.7	•	72.3	245.1	•
	Bottom	32.6	84.4	84.3	57.8	130.4	134.2
Depth (µm)	Left	•	25.3	21.7	56.6	•	•
	Center	63.9	38.5	22.9	53.9	44.6	44.5
	Right	•	48.2	39.8	50.6	•	•
Side of crack	Line types	Verticality	Oblique & Verticality	Verticality	Verticality	Oblique	Oblique
	Surface Shape	Flat	Flat	Flat	Flat	Flat	Flat
Underside of crack	Line types	Horizontal	Horizontal & Puddle	Horizontal	Horizontal	Puddle	Puddle
	Surface shape	Flat	Flat	Flat	Flat	Smooth	Smooth

Figure S10. It was found that all the measured cracks showed the color of the lower layer, and the thickness of the paint layer for each crack showed a difference of up to 40 µm. Cracks with a soft underside or side surface were observed, but the depth was generally very shallow. In particular, the shape of cracks with shallow widths was generally rectangular.

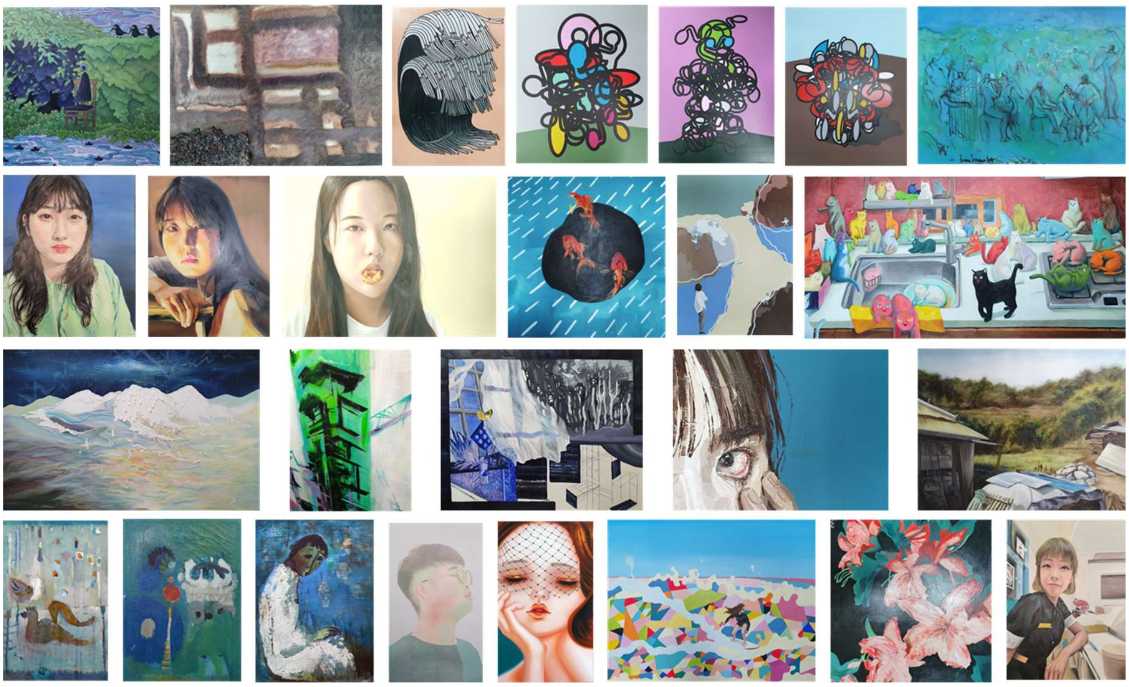

Figure S11. A list of oil painting works borrowed to analyze the surface features and cracks of oil paints in this study. Although a common material called oil paint was used, the time of production, the artist, and the production technique differs for each work. In order to analyze the surface characteristics of oil painting works and the three-dimensional shape of cracks, several works were borrowed to collect scientific data.
